# Data Resource Profile: The Education and Child Health Insights from Linked Data (ECHILD) Database

**DOI:** 10.1093/ije/dyab149

**Published:** 2021-11-11

**Authors:** Louise Mc Grath-Lone, Nicolás Libuy, Katie Harron, Matthew A Jay, Linda Wijlaars, David Etoori, Matthew Lilliman, Ruth Gilbert, Ruth Blackburn

**Affiliations:** 1 University College London, Institute of Health Informatics, London, UK; 2 Centre for Longitudinal Studies, University College London, Institute of Education, London, UK; 3 University College London, Great Ormond Street Institute of Child Health, London, UK

**Keywords:** **Key words: **Child health, adolescent health, education, administrative data, linked data, social care

Key Features

- The Education and Child Health Insights from Linked Data (ECHILD) Database was established to enable large-scale, longitudinal research that explores inter-relationships across the domains of health, education and social care in childhood and adolescence.
- The ECHILD Database brings together administrative data from Hospital Episode Statistics (HES) and the National Pupil Database (NPD) for all children and young people aged 0–24 years in England who were born between 1 September 1995 and 31 August 2020. In total, it includes linked HES and NPD records for approximately 14.7 million individuals.
- The ECHILD Database includes information about hospital admissions, accident and emergency attendances, outpatient appointments and deaths. It also includes information about pupil characteristics, educational outcomes and use of children’s social care services.
- The data sources included in the ECHILD Database are collected on a regular, ongoing basis and collated to create longitudinal, individual-level records. The baseline year of data collection varies, with some social care data included from 1995, health data from 1997 and education data from 2001.
- The ECHILD Database will be available to accredited researchers in 2021 by applying to the data providers (Department for Education and NHS Digital). To discuss opportunities for collaboration, please contact the ECHILD project team at [ich.echild@ucl.ac.uk].

## Data resource basics

The Education and Child Health Insights from Linked Data (ECHILD) Database is a linkable collection of longitudinal, administrative datasets from the domains of health, education and social care for a whole population-based cohort of children and young people in England. The ECHILD Database is de-identified. It does not include any information that could be used to directly identify a person, such as names, addresses, postcodes or dates of birth. Access and outputs are strictly controlled and re-identification of individuals is not permitted. Ethical approval for the ECHILD project was granted by the National Research Ethics Service (17/LO/1494), NHS Health Research Authority Research Ethics Committee (20/EE/0180) and UCL Great Ormond Street Institute of Child Health’s Joint Research and Development Office (20PE16).

The ECHILD Database was created as part of the ECHILD project, a research study led by University College London in partnership with National Health Service (NHS) Digital and the Department for Education (DfE). The aim of the ECHILD project is to explore the inter-relationship between health and education outcomes for children and young people in England, particularly for vulnerable groups, such as those with adverse birth characteristics and chronic health conditions. For example, we are using the ECHILD Database to explore the impact of disruptions to health and education services during the COVID-19 pandemic on hospital attendances for children and young people in England. However, the ECHILD Database can be used for a wide variety of analyses, provided they benefit education, health and social care. The creation of the ECHILD Database was funded by ADR UK (Administrative Data Research UK), an Economic and Social Research Council (part of UK Research and Innovation) programme.

The ECHILD Database includes all children and young people in England who: (i) were born between 1 September 1995 and 31 August 2020; and (ii) had any record in Hospital Episode Statistics (HES) or the National Pupil Database (NPD). HES and NPD are national administrative datasets covering the whole population of children and young people in England who receive state-funded health, education or social care services, including those who were born outside the country. HES contains records for all hospital activity that is provided or paid for by the NHS in England, including births, inpatient admissions, outpatient appointments and accident and emergency (A&E) attendances.^1^ NPD contains records related to state-funded education and use of children’s social care services.^2^ In total, the ECHILD Database includes linked HES and NPD records for approximately 14.7 million individuals. Information is included, where available, from birth up to an individual’s 25^th^ birthday, as this is widely considered the end of adolescence.^3^

**Figure 1:**
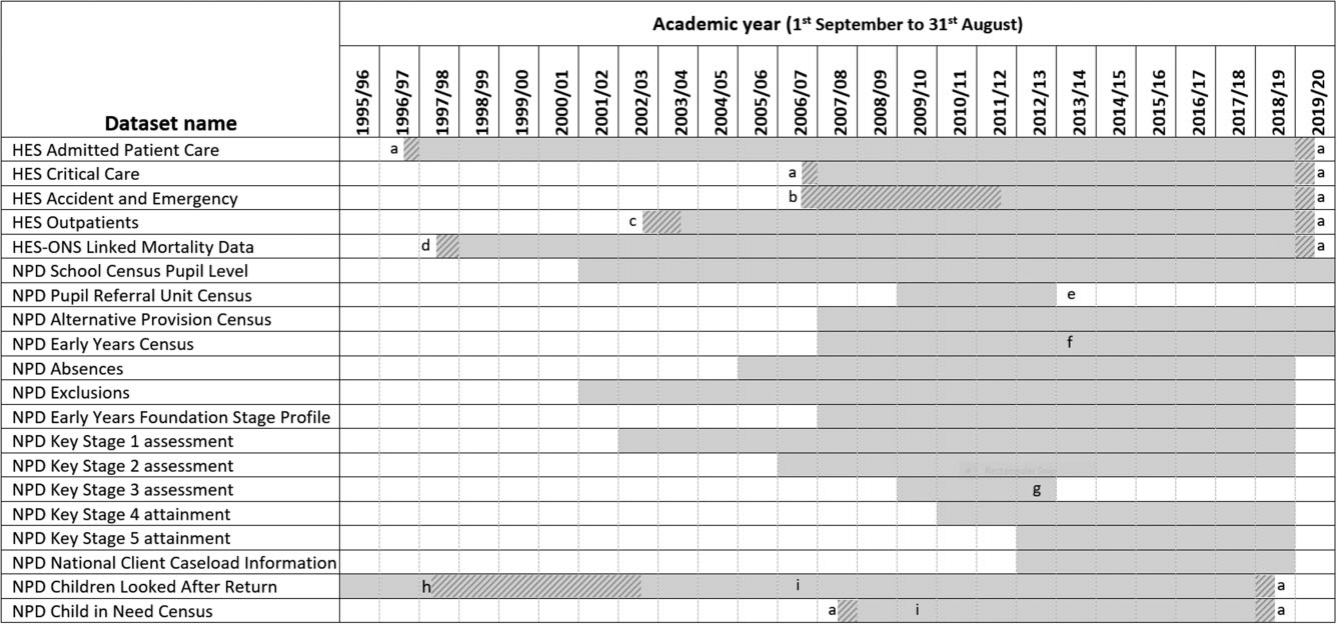
Overview of data available in the Education and Child Health Insights from Linked Data (ECHILD) Database, by academic year: data available in the ECHILD Database as of May 2021. Data availability may change over time. Grey hatching indicates partial coverage. HES, Hospital Episode Statistics; ONS, Office for National Statistics; NPD, National Pupil Database. A. Partial coverage of an academic year, as NPD social care data and HES data are collated by financial year (1 April to 31 March). B. Partial coverage, as HES Accident and Emergency data were experimental and did not have full national coverage. C. Partial coverage, as HES Outpatient data were experimental and did not have full national coverage. D. Partial coverage of an academic year, as ONS mortality data were first linked to HES in January 1998. E. The Pupil Referral Unit Census was subsumed in the School Census Pupil Level from 2013/14. F. The Early Years Census included 3- and 4-year-olds between 2007/08 and 2012/13. From 2013/14, it includes 2- to 4-year-olds. G. Key Stage 3 assessments ceased after 2012/13. H. Partial coverage of population, as between 1 April 1998 and 31 March 2003, Children Looked After Return data were only collected for a one-third sample (i.e. children with a day of birth divisible by 3). i. Linkage between education and social care modules of NPD began in April 2006 for the Children Looked After Return and in April 2009 for the Child in Need Census

HES and NPD are well-established datasets that collect and collate information on a regular, ongoing basis to create individual-level, longitudinal records. Data within HES and NPD are organized into separate modules and the earliest year of collection (baseline) varies for each module (Figure 1); for example, pupil characteristics are available from 2001 and hospital admissions data are available from 1997. When the ECHILD Database was created in November 2020, all HES and NPD data that were available were included. The most recent time period during which data were available varied by data module due to differences in data collection periods and lags between data being collected and made available for research (Figure 1); for example, information related to social care was available up to 31 March 2019, whereas information related to hospital admissions was available up to 31 March 2020. Currently, the ECHILD Database includes information for young people up to the age of 25 years; however, it will be further updated to include more recent HES and NPD data as they become available, thereby enabling follow-up of health records into adulthood.

## Data collected

The HES data included in the ECHILD Database contains information from hospital records for all NHS patients in England, including demographics and standardized codes for diagnoses, symptoms and procedures relating to the care they have received.^4^ HES data are collected by NHS Digital from care providers and are curated on an ongoing basis in four modules related to different aspects of hospital care. The HES Admitted Patient Care (APC) module records hospital admissions and treatment that requires the use of a hospital bed, including births and day cases.^5^ The HES Critical Care module records treatment for the subset of admitted adult patients where constant support and monitoring in adult designated wards is required to maintain at least one organ (i.e. an intensive care or high dependency unit). The HES Accident and Emergency (A&E) module records attendances at A&E departments, including some walk-in centres and minor injury units. The HES Outpatients module records all outpatient appointments at English NHS hospitals and the independent sector (if commissioned by the NHS), regardless of whether the appointment was attended or not. Since January 1998, HES data have been routinely linked to Office for National Statistics (ONS) mortality data.^6^ This mortality information is also included in the ECHILD Database. Table 1 illustrates the range of health-related HES variables that are included in the ECHILD Database.

**Table 1:** Selection of key health-related variables from Hospital Episode Statistics included in the Education and Child Health Insights from Linked Data (ECHILD) Database

HES data module	Demographic	Clinical	Geography
Admitted Patient Care	Age Sex Ethnic group	Diagnoses (up to 20)^a^ Operations (up to 24)^a^ Operation dates Consultant specialty	Middle Super Output Area Local authority Clinical commissioning group Index of multiple deprivation decile
Critical Care	N/A	Critical care start date Critical care discharge date Number of days of support by organ group Discharge destination	N/A
Accident and Emergency	Age Sex Ethnic group	Type of attendance Mode of arrival Treatments (up to 12)^a^ Duration to treatment	Local authority Clinical commissioning group Index of multiple deprivation decile
Outpatients	Age Sex Ethnic group	Type of appointment Outcome of appointment Medical staff type seeing patient Duration of elective wait	Local authority Clinical commissioning group Index of multiple deprivation decile
ONS Linked Mortality	Sex	Month and year of death Underlying cause of death	N/A

HES = Hospital Episode Statistics; ONS = Office for National Statistics; N/A, not available.

a Information on diagnoses, treatments and procedures for each episode of care is recorded by clinical coders based on patient care records and/or discharge summaries using standardized codes. In the Admitted Patient Care, Critical Care and Outpatient modules, diagnoses are recorded using the International Classification of Disease (ICD) version 10, and treatments and procedures are recorded using the Office of Population Censuses and Surveys (OPCS) version 4. In the Accident and Emergency module, bespoke codes are used to record diagnoses and treatments^14^; however, these are much more limited than ICD-10 and OPCS-4 codes.

The ECHILD Database also contains information related to education and children’s social care from the NPD. The NPD is made up of several modules that are collected by the DfE from schools, local authorities and examination awarding organizations on an ongoing and statutory basis.^7^ The NPD includes four education census modules that collect information about the characteristics of pupils in different educational settings (Table 2). These pupil characteristics include age, gender, ethnicity, special educational needs (SEN), and free school meals (FSM) eligibility. Pupils are eligible for FSM if their parents are in receipt of certain mean-tested benefits, and eligibility is often used in research as a proxy for income disadvantage.^8^ The education census modules do not collect information for the estimated 7% of children in England enrolled in a private school,^9^ 0.1% in a hospital school (authors’ calculation from DfE statistics)^10^ or 0.7% who are home educated (authors’ calculation from Office of the Schools Adjudicator statistics).^11^ These children will have incomplete educational information in the ECHILD Database; however, the vast majority of children in England (at least 92%) will have some educational information recorded in the ECHILD Database. The NPD also includes information on educational outcomes with modules related to absences, exclusions (whereby a child is temporarily suspended or permanently expelled from school), attainment in national assessments and examinations, and participation in post-16 education. In England, participation in education is compulsory up to age 18; however, after the age of 16 this can include a combination of education, apprenticeships, training and part-time work.

**Table 2:** Overview of education and social care modules from National Pupil Database included in the Education and Child Health Insights from Linked Data (ECHILD) Database

NPD data module	Who is information collected for?	Examples of information that is collected
Early Years Census^a^	All 2- to 4-year olds in state-funded early years care and education	Age; gender; ethnicity; SEN
School Census Pupil Level ^a^	All pupils in state-maintained educational settings, excluding hospital schools	Age; gender; ethnicity; SEN; FSM eligibility; language
Pupil Referral Unit Census^a^	All pupils in a PRUs (non-mainstream schools maintained by the state)	Age; gender; ethnicity; SEN; FSM eligibility; language
Alternative Provision Census^a^	All pupils in non-mainstream, non-maintained educational settings for whom the state are covering tuition costs	Age; gender; ethnicity; SEN; FSM eligibility
Absences	All pupils aged 4–15 in state-maintained educational settings, excluding boarding pupils	Number of absences; numbers that were authorized and unauthorized
Exclusions	All pupils in state-maintained educational settings	Number of fixed period exclusions; number of permanent exclusions
Early Years Foundation Stage Profile	All children at the end of the Early Years Foundation Stage of education (age 5)	Early years practitioner assessment scores
KS1, 2 and 3 assessments	All children at the end of KS1 (age 7), KS2 (age 11) and KS3 (age 14)	Teacher assessments scores
KS4 qualifications	All pupils in KS4 (age 15–16), including those in private schools	Entry for and attainment in GCSE and equivalent qualifications
KS5 qualifications	All pupils in KS5 (age 16–18), including those in private schools	Entry for and attainment in A-level and equivalent qualifications
National Client Caseload Information System	All young people aged 16–19 and young people aged 20–25 who have an SEN or disability	Post-16 activity; not in education, employment or training indicator
Children in Need Census	Referrals to children’s social care and all children in need	Referral date; category of need; start date of child protection plan
Children Looked After Return	All children who are looked after	Placement start date; type of placement setting; legal basis for placement

NPD, National Pupil Database; PRU, pupil referral unit; SEN, special educational needs; FSM, free school meals; GCSE, General Certificate of Secondary Education (national examinations taken by students at the end of compulsory education); KS, Key Stage.

a The School Census Pupil Level module is collected on a termly basis in October (Autumn census), January (Spring census) and May (Summer census). The other education census modules are collected in January only.

The NPD has two social care modules which are both included in the ECHILD Database. The Children in Need (CIN) census collects information related to children referred to social care services and those identified as needing additional help from those services to support their health or development.^12^ The CIN census includes child characteristics (e.g. age, ethnicity, gender), as well as details of social care referrals, reviews, assessments and use of child protection plans (for children assessed to be at risk of serious harm). The Children Looked After Return (CLA) includes information related to children in care, who are referred to as looked-after children in the UK.^13^ The CLA contains information related to child characteristics, placements in out-of-home care and adoptions.

## Production of the ECHILD database

HES and NPD include a pseudonymized identifier that allows records relating to the same individual to be linked across modules and over time (HESID_15_ and anonymized Pupil Matching Reference (aPMR),^16^ respectively). As there is no common pseudonymized identifier in HES and NPD, the ECHILD Database was created by NHS Digital by linking records based on identifiable information (specifically, name, date of birth, sex and chronology of postcode). According to N Libuy, PhD (written communication, February 2021), initial assessments of linkage quality in the ECHILD Database indicate very high linkage rates between HES and NPD records, with linkage rates improving from 94% for children born in 1996/97 to 98% linkage for children born in 2004/05.

Full details of the linkage process to create the ECHILD Database have previously been published.^17^ Briefly, the DfE extracted identifiers and associated aPMRs from NPD to separate them from attribute information about individuals’ education and social care records. This information was securely transferred to NHS Digital. Similarly, at NHS Digital, identifiers and associated HESIDs were extracted from HES, separating them from attribute information about individuals’ health and use of services. Deterministic linking algorithms were used by NHS Digital to link HESIDs and aPMRs and create a pseudonymized bridging file with indicators of link quality but no identifiable information. NPD and HES attribute data and HESID-aPMR bridging files were then separately transferred to a trusted research environment, the ONS Secure Research Service (ONS SRS),^18^ and collated to create the ECHILD Database. The ECHILD Database is de-identified: it does not include any information that could be used to directly identify a person, such as names, addresses, postcodes or dates of birth. The pseudonymized identifiers it contains (HESID and aPMR) cannot be linked to real-world identifiers (such as, NHS or National Insurance numbers) by researchers, including the ECHILD project team.

## Data resource use

The ECHILD project is currently using the ECHILD Database for large-scale, longitudinal research that explores inter-relationships across the domains of health, education and social care in childhood and adolescence. For example, we are exploring the relationship between gestational age at birth, chronic health conditions, school attainment and special educational needs (SEN) in later childhood. Previous research using linked administrative data in Scotland has indicated a dose-response relationship between gestational age at birth and risk of SEN.^19^ The creation of the ECHILD Database means it is now possible to explore whether there is a similar relationship for children in England and how this association is related to chronic health conditions. These results will be useful for policy makers and service providers; for example, for estimating future need for SEN support in schools based on birth characteristics of the population. The ECHILD project is also using the ECHILD Database to explore the impact of disruptions to health and education services during the COVID-19 pandemic on hospital attendances for children and young people in England. In particular, it focuses on whether children with additional needs (e.g. with a chronic health condition, in care or receiving SEN support) were more affected by service disruptions than their peers. A full list of publications from the ECHILD project is available at: [https://www.ucl.ac.uk/child-health/echild].

## Strengths and weaknesses

Health and education are strongly interconnected for children and young people. For example, children with chronic health conditions have higher rates of school absence and poorer school performance than their peers.^20^ Acknowledging these inter-relationships, policy makers have called for greater collaboration between service providers in these domains.^21^ HES and NPD are well-established administrative datasets for health and education in England. These datasets act as an evidence base to inform policy: they are used to produce national statistics by government departments and for wider research purposes by the academic community. However, the lack of a common identifier in these administrative datasets has limited the potential for wide-scale analysis across domains. A strength of the ECHILD Databases is that by linking data across these domains, it presents a unique and valuable opportunity to explore how children’s health affects their education, and how their education affects their health. A further strength of the ECHILD Database is that it brings together de-identified information from long-standing, national administrative datasets. These constituent datasets include longitudinal, individual-level information for a large, whole-population-based cohort of children and young people. The large sample size (14.7 million individuals) and long follow-up period (up to 25 years) in the ECHILD Database will enable research into long-term outcomes and rare exposures. The constituent datasets are also well documented by data owners^1^^,^^22^ and the research community.^4^^,^^5^^,^^12^^,^^13^^,^^23^^,^^24^ This means that details about how information in the datasets is collected, what variables they contain and how coding has changed over time, for example, are readily available to researchers. The key strength of the ECHILD Database is that the use of the data is safeguarded by being de-identified and accessible only via a trusted research environment, the ONS SRS. In the ONS SRS, there is strict monitoring of data access and use and scrutiny of outputs, including any tables, graphs or figures. Researchers using the ONS SRS have to undergo training in governance procedures and sign data access agreements that prohibit any attempt to re-identify individuals. Such attempts could lead to data access for their institution being revoked.

The main limitation of the ECHILD Database is that administrative datasets are not collected for research purposes. This may have implications for the type of research that can be carried out and how findings are interpreted.^25^ For example, HES data are primarily used for payment purposes (i.e. care providers are reimbursed from NHS England through the ‘Payment by Results’ system), and so there may be differences in the quality and completeness of the information that is recorded based on the impact it has on payment. A further limitation is that the range of information collected in HES and NPD varies over time, and earlier years of data and linkage are considered to be of lower quality. However, both datasets are subject to data quality assurance checks at the point of submission to DfE or NHS Digital and are considered of high enough quality to produce national statistics. There are also issues related to missing data in non-mandatory HES variables; for example in the HES Outpatient module, primary diagnosis is not recorded for 95% of records.^4^ These limitations are well documented, but it is important that researchers using the ECHILD Database familiarize themselves with the constituent datasets to understand the potential limitations and caveats of their proposed analyses.

## Data resource access

The ECHILD Database will be available to accredited researchers in 2021 by applying to the data providers (DfE and NHS Digital). Further documentation about the ECHILD Database, including an introductory user guide^17^ and data catalogue, is available at: [ucl.ac.uk/child-health/echild]. To discuss opportunities for collaboration with the ECHILD project team, please contact: [ich.echild@ucl.ac.uk].

## Funding

This work is supported by ADR UK (Administrative Data Research UK), an Economic and Social Research Council (part of UK Research and Innovation) programme [grant number ES/V000977/1]. This research was also supported in part by the NIHR Great Ormond Street Hospital Biomedical Research Centre and Health Data Research UK [grant number LOND1], funded by the UK Medical Research Council and eight other funders. This research benefits from and contributes to the NIHR Children and Families Policy Research Unit, but was not commissioned by the National Institute for Health Research (NIHR) Policy Research Programme. R.G. and R.B. are in part supported by the National Institute for Health Research (NIHR) Children and Families Policy Research Unit. The views expressed are those of the authors and not necessarily those of the NIHR or the Department of Health and Social Care. R.B. is supported by a UKRI Innovation Fellowship funded by the Medical Research Council [grant number MR/S003797/1]. K.H. is funded by Wellcome Trust [grant number 212953/Z/18/Z] and NIHR [grant number 17/99/19]. M.J. is funded by the Medical Research Council through the UCL-Birkbeck Doctoral Training Partnership.

## Author contributions

LM-L wrote the manuscript, with critical input from all authors. All authors approved the final manuscript.

## Conflict of interest

None declared.
